# Characterization of Glass Insulating Thick Films with Ag Conductors for Multilayer Packages

**DOI:** 10.3390/ma14030494

**Published:** 2021-01-21

**Authors:** Jun Fang, Renli Fu, Xiguang Gu, Xinyao Zhang, Guojun Li

**Affiliations:** 1 College of Materials Science and Technology, Nanjing University of Aeronautics and Astronautics, Nanjing 211106, China; renlifu@nuaa.edu.cn (R.F.); guxiguang_tmri@163.com (X.G.); zxy_nuaa_cl@163.com (X.Z.); liguojun@nuaa.edu.cn (G.L.); 2 Nanjing Miko Electronic Technology Co., Ltd., Nanjing 211235, China

**Keywords:** thick insulating film, screen-print, Ag conductor, multilayer package

## Abstract

In this paper, an insulating film was successfully prepared by sintering 35 wt % CaO-15 wt % Al_2_O_3_-10 wt % B_2_O_3_-40 wt % SiO_2_ glass at 875 °C. After sintering, the main component of the insulating film was glass-ceramics. The main crystal phase was CaAl_2_Si_2_O_8_, and the crystallization activation energy was 189.76 kJ/mol. After preparing the insulating film, its color turned yellow, and the diffusion of Ag was found by XPS and XRD data. When the temperature increased to 875 °C, the color of the insulating film became lighter, and the silver content decreased. The adhesion of the multilayer structure could reach 875 N. The dielectric constant of the insulating film in the multilayer structure was approximately 5, and the dielectric loss was 0.0011. After sintering, the dielectric strength of the insulating film could reach 13.11 kV/mm, which fully meets the requirements of a complex packaging structure.

## 1. Introduction

With the requirements of electronic device function integration and small size, the density and quantity of electronic components on packaging substrate are increasing rapidly. To package more electronic components in a smaller area, more complex multilayer structure packaging modes, such as three-dimensional (3D) packaging technologies, have been developed [1]. The performance requirements of the substrate are also higher, and the circuit density increases and becomes more complex.

Thick film technology is a process of forming metal circuits onto the surface of the ceramic substrate by screen printing, which is a direct printing method. In screen printing, a printable paste is printed onto the substrate with the help of a screen mask and then sintered at a high-temperature of 500–1000 °C to form a graphic circuit on the ceramic surface [2,3]. This technology can realize the fabrication of a multilayer circuit on the ceramic substrate. The realization of the multilayer structure will reduce the volume and area of the substrate effectively [4,5,6,7].

The key to constructing the multilayer structure is to realize two points, namely, the matching between metal and the insulating dielectric layer and the insulation in the different layers of the metal circuit. Silver (Ag) and Ag alloy are usually selected for metal circuits because of their high conductivity and direct sintering in the air atmosphere. However, Ag, as a conductor in a thick-film multilayer structure, also experiences several challenges. Ag will diffuse into the substrate material, thereby resulting in the degradation of performance [8,9,10]. Glass ceramic is generally used as an insulating material, which can adjust the coefficient of thermal expansion and control crystallization so as to control the diffusion of metal in the interior [4,5]. The CaO-Al_2_O_3_-B_2_O_3_-SiO_2_ glass is an aluminosilicate glass system, which has been fully studied. Because of its low dielectric constant (4–5) and low dielectric loss (~0.002), it is an ideal electronic packaging material and widely used in low-temperature co-fired ceramic (LTCC) substrate. In addition, the system does not contain lithium, sodium, potassium and other alkali metal elements, so it is more suitable to construct a multilayer structure, mainly because alkali metal elements are easy to diffuse between layers. The coefficient of thermal expansion of the glass system is ~9 ppm/K, which is matched with the alumina substrate (6.5–7.5 ppm/K), which can make the multilayer structure more reliable and more suitable for electronic packaging [11,12,13,14,15]. Based on the good electrical properties of the glass and the shrinkage matching of co-firing with Ag is suitable for LTCC technology, it is achievable for forming a new thick film printed multilayer structure with Ag as an insulating film. Although it is possible to construct a multilayer structure with LTCC technology, it requires high technology. Herein, the thick film method provides a convenient and efficient method to print glass insulating film and Ag film to construct a multilayer structure. Taking advantage of this method, the multilayer structure based on glass and Ag is easier to prepare the complex circuit on the small size substrate. Furthermore, it can be used in light-emitting diode (LED) modules and power modules, which require a complex connection among electronic components [6,7].

However, few studies have focused on the application of glass in multilayer Ag as an insulating film. Whether there are differences in the phase and crystallization mode of its formation and how to match with the Ag layer are new problems to be faced. In addition, if there is silver diffusion in the LTCC substrate, whether there is silver diffusion in the dielectric layer. In addition, because the film thickness is thinner than that of ceramics, it is worth to study the specific electrical properties.

In this paper, the thick film method was applied to create the insulating film between two Ag films based on CaO-Al_2_O_3_-B_2_O_3_-SiO_2_ glass on the alumina substrate. The composition, crystalline properties, and surface morphology of the insulating film after sintering are discussed. Finally, the multilayer structure is formed, and the electrical and mechanical properties of the insulating film are investigated.

## 2. Materials and Methods

### 2.1. Preparation of Insulating Thick Films

The composition of CaO-Al_2_O_3_-B_2_O_3_-SiO_2_ glass was selected in this study. Reagent-grade raw materials, CaCO_3_ (AR, Sinopharm, Nanjing, China), Al_2_O_3_ (AR, Sinopharm, Nanjing, China), and SiO_2_ (AR, Sinopharm, Nanjing, China), were mixed with H_3_BO_3_ (AR, Sinopharm, Nanjing, China) (Table 1). The resulting powders were melted at 1400 °C for 2 h and then were quenched in deionized water. The mechanical crushing of the quenched glass was performed to form a glass frit. The insulating pastes were prepared by mixing the 25 wt % organic solvent composed of terpineol and ethyl cellulose with 75 wt % glass.

The sample had a laminated structure on the surface of the alumina substrate. With the help of screen-printing technology, the insulating paste was printed between the conductive layers, which were formed by Ag paste (F14015, 3Focus Co., Guangzhou, China) and sintered step-by-step to form a three-layer structure. The Ag layers were sintered at 850 °C for 15 min, whereas the insulating film was sintered at 825–900 °C for 30 min.

### 2.2. Fabrication of Insulating Thick Films

**Figure 1:**
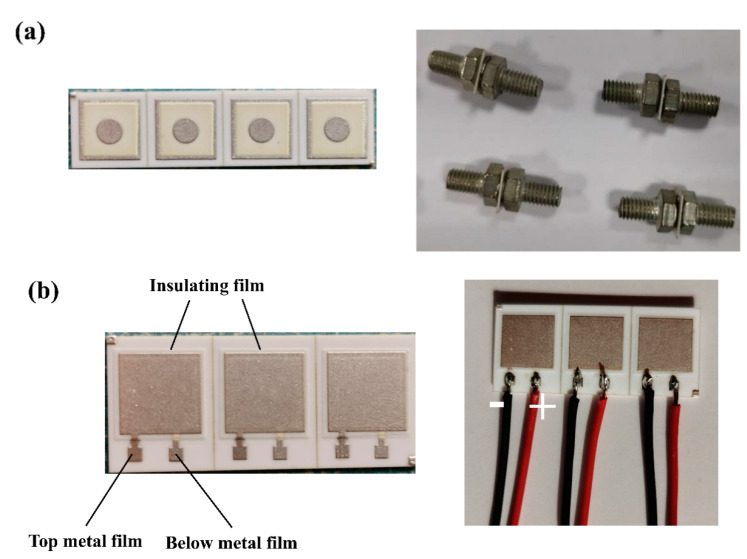
Mechanical and electrical test patterns of the samples. (**a**) Mechanical test patterns and samples. (**b**) Electrical test patterns and samples.

The surface and cross-section of the thick insulating films were investigated by a scanning electron microscope (SEM, Hitachi Regulus 8100, Tokyo, Japan) equipped for energy-dispersive spectroscopy (EDS, Bruker Quantax70, Tokyo, Japan). The sample was treated in liquid nitrogen at low-temperature to make it brittle, and then a cross-section of the sample was obtained by fracture. Approximately 1 g glass powder was made into a small cylinder with a diameter of 8 mm and a thickness of 10 mm to measure the coefficient of thermal expansion of glass. The coefficient of thermal expansion (CTE, DIL 402PC, NETZSCH Co., Selb, Germany) was measured at 20–900 °C. The surface of the samples was observed by the LEICA camera. The crystallization temperature for the samples was determined by differential scanning calorimetry (DSC, STA 409 PC/PG, NETZSCH Co., Selb, Germany) with different heating rates. The valence states of elements in the insulating film that was made of glass ceramics were measured by x-ray photoelectron spectroscopy (XPS, ESCALAB250Xi, Thermo Fisher Scientific, Waltham, MA, USA). The adhesion of the top Ag film was measured by using the pull-off test method. The test patterns and samples are shown in Figure 1a. The Ag-plated M6 brass screws were soldered to both sides of the samples to complete the next test. The equipment jaws (SANS universal testing machine, CMT5105, Shenzhen, China) moved away with a speed of 5 mm/min until the sample was pulled off.

The electrical parameters of the samples were measured. The main attention was paid to dielectric strength measurement. A high voltage source (Tonghui TH9320 HIPOT TESTER, Suzhou, China) with a frequency of 50 Hz and a voltage rate of 50 V/s until breakdown was used. Other electrical parameters included capacity and dielectric loss through the insulating films and were measured by Tonghui TH2817B + LCR Meter (Suzhou, China). Figure 1b shows the test pattern of the electrical parameters. Table 2 describes the details of the test specimens.

## 3. Results and Discussion

### 3.1. Glass Selection for Insulating Thick Films

**Figure 2:**
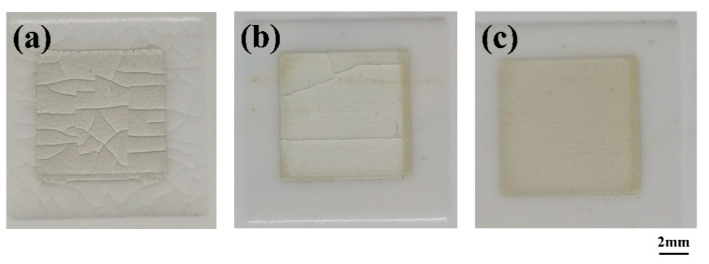
Surface morphology of insulating film on the surface of the Ag layer, the sample were sintered at 875 °C for 30 min after screen-printing. (**a**) Cracks appear in the film made of insulating pastes 1 (paste 5 is the same), (**b**) few cracks appear in the film made of paste 2, and (**c**) uniform film without cracks were made of insulating pastes 3 (paste 4 is the same).

The insulating pastes made of glass were screen-printed on the Ag layer of the ceramic substrate and sintered at 875 °C for 30 min to form thick insulating films. However, differences were observed in the surface morphology of the insulating films. Some cracks appeared in the film made of insulating pastes 1 and 5, whereas the film made of pastes 3 and 4 was uniform. No obvious crack was found (Figure 2). Defects, such as cracks, can affect the insulation performance of the upper and lower metal circuit layers.

**Figure 3:**
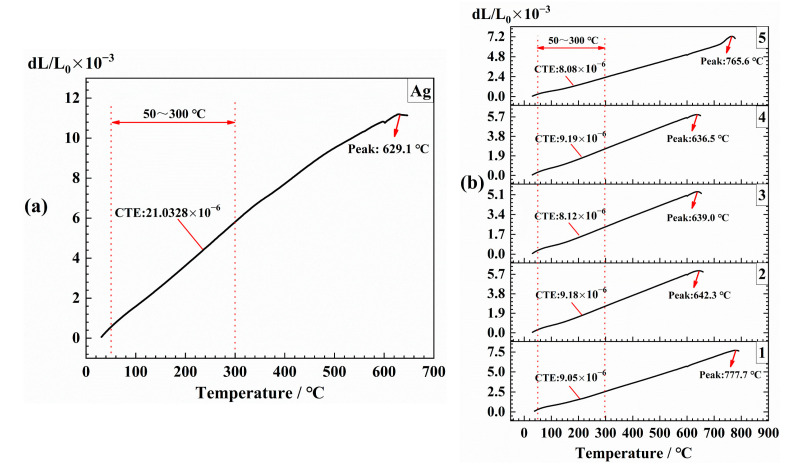
Coefficient of thermal expansion (CTE) of the sample is in a range from 20 °C to 300 °C, and the softening point was tested, and the CTE and softening point of each sample were marked with a red line. (**a**) Ag, (**b**) No. 1–5 glass-ceramics.

The difference between the Ag layer formed after the sintering of Ag paste and the metallic Ag was not large, that is, about 21.03 × 10^−6^ at range 50–300 °C (Figure 3a). However, in Figure 3b, the CTE of glass–ceramic had a slight difference, which was approximately 8~9 × 10^−6^. A big difference between the Ag and insulating film was observed, but the insulation film made of pastes 3 and 4 was relatively uniform (no obvious crack), thereby indicating that there was no necessary relationship between the CTE and the appearance of cracks. However, a big difference was observed in the softening point among different glasses (Figure 3). The softening point of Ag was 629.1 °C (Figure 3a), and the No. 3 and No. 4 were the closest at 640 °C (Figure 3b), which could promote the elimination of cracks. The softening point of No. 2 was slightly higher than 640 °C, but it was also close to the softening point of Ag, and the cracks were much less than those of No. 1 and No. 5. It was not difficult to see that, compared with the CTE, the insulation layer with a softening point close to the Ag layer was easier to obtain uniform surface morphology, which had a positive effect on controlling the generation of cracks.

### 3.2. Phase Analysis and Composition of Glass for Insulating Thick Films

Based on the uniform, crack-free surface morphology, No. 4 glass was selected to study the properties of insulating films sintered at 825–900 °C.

**Figure 4:**
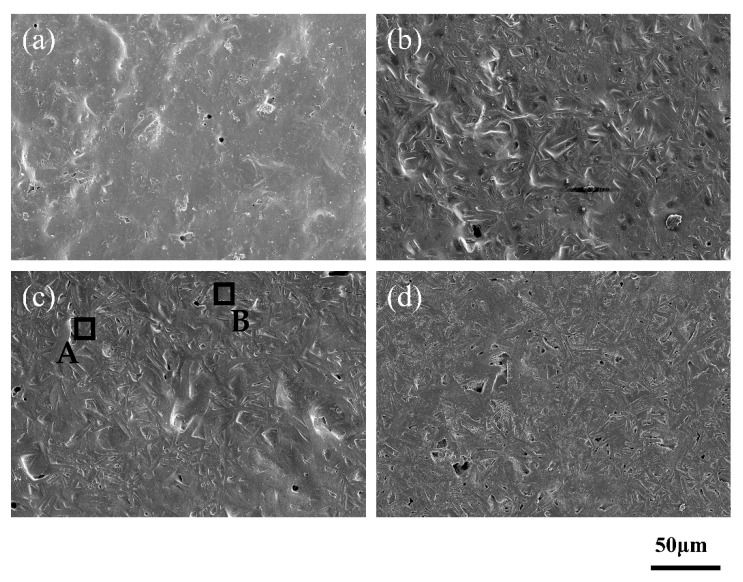

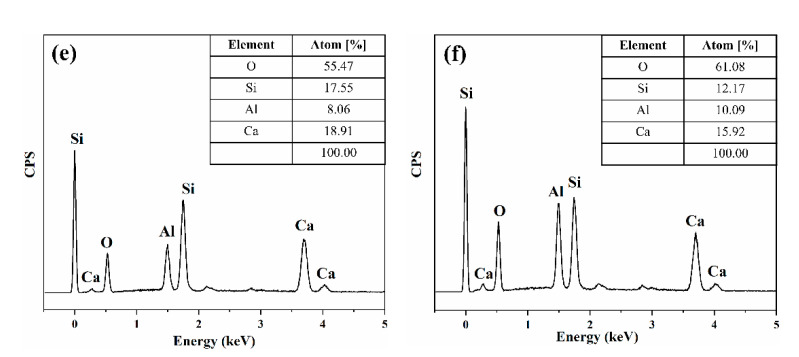
Surface morphology of No. 4 glass sintered at different temperatures. (**a**) 825 °C; (**b**) 850 °C; (**c**) 875 °C; (**d**) 900 °C. The energy-dispersive spectroscopy (EDS) spectra of the crystals in the black frame are shown in (**e**) region A and (**f**) region B.

With the increase of temperature, the crystal content on the surface of the insulating film increased, and the crystal gradually became finer on the larger one (Figure 4a–d.) However, the EDS spectra show that the main elements in the crystal were O, Si, Al and Ca, but the relative contents were different. In the beginning, the content of Al was less than that of Si. When the temperature increased, the content of Al increased and the content of Si decreased (Figure 4e,f).

**Figure 5:**
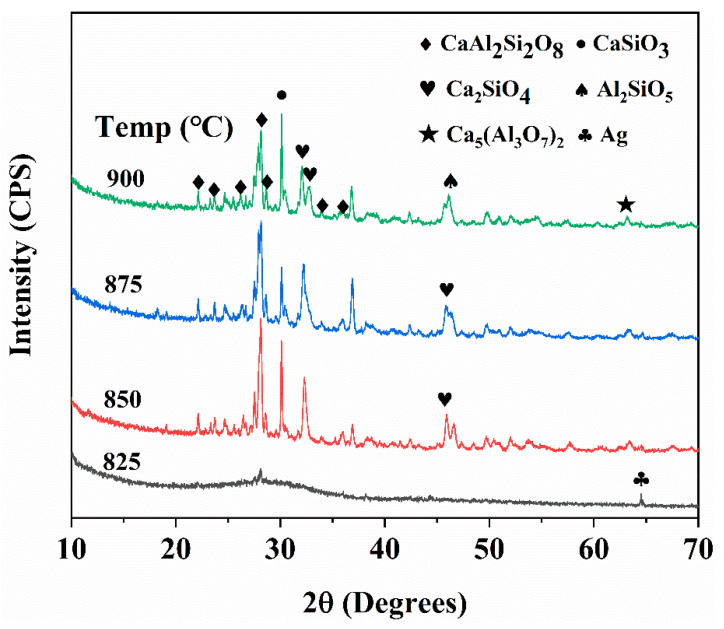
XRD pattern of No. 4 insulating film sintered at 825–900 °C for 30 min.

The XRD patterns of insulating film glass sintered at 825–900 °C for 30 min on Ag film are shown in Figure 5. At 825 °C, most of them were in a glass state with only a few crystallization peaks. This was consistent with the surface morphology in Figure 4a. There was still a glass phase, and there was no obvious crystalline phase on the surface. The obvious crystallization began from 850 °C, and the major XRD peaks were identified as CaSiO_3_ and CaAl_2_Si_2_O_8_. Although both A and B regions contain the same elements in the energy spectrum, it may have been caused by the fusion of the two kinds of grains. It can be seen that the content of Al and Si in the fine crystal tended to be the same (B region), which should be CaAl_2_Si_2_O_8_. In addition, there were the peaks of CaSiO_3_, Ca_2_SiO_4_, Al_2_SiO_5_, Ca_5_(Al_3_O_7_)_2_. Although the insulating film and LTCC used the same kind of glass, they had different crystal phase compositions, which could be mainly due to the different manufacturing processes [16].

**Figure 6:**
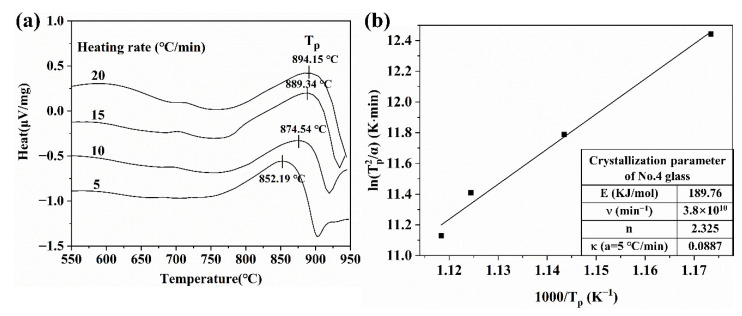
(**a**) Differential scanning calorimetry (DSC) crystallization peaks (*Tp*) at heating rates of 5, 10, 15, and 20 °C/min. (**b**) Kissinger plots (*ln (*$T_{p}^{2}$*/α)* vs. 1000/*Tp*) at heating rates of 5, 10, 15, and 20 °C/min. The table inside has the crystallization parameters of the No. 4 glass.

The crystallization of No. 4 glass was investigated using DSC. As shown in Figure 6a, although many crystalline phases were found in the XRD pattern, only one obvious crystallization peak was found in the DSC curve. This phenomenon may be due to the close crystallization temperature of each crystal phase that the crystallization peaks overlapped. The crystallization kinetics of No. 4 glass was studied by changing the heating rate of 5, 10, 15, and 20 °C/min during the DSC process. The crystallization kinetic parameters of No. 4 glass were calculated by Arrhenius equation [17], Kissinger equation, and Augis–Bennett equation [18], which can be expressed as follows:

(1) k=vexp(−E/RT),

(2) $$\ln((T_{p}^{2})/\alpha )=E/(RT_{p})+\ln(E/R)-\ln v,$$

(3) $$n=2.5/FWHM\times (RT_{p}^{2})/E$$

where *k* is the reaction rate constant, ν is the frequency factor, *E* is the activation energy, *R* is the gas constant, *T* is temperature, *α* is the DSC rate, *n* is the crystallization index, and *FWHM* is the half-height temperature wideness of the maximum exothermic peak of DSC.

In Figure 6a, the glass exhibited different crystallization peak temperatures at various heating rates. Equation (2) shows that *ln ($T_{p}^{2}$/α)* is proportional to *1/Tp*, and the relationship between them is shown in Figure 6b. Calculating the crystallization kinetic parameters of No. 4 glass from Equations (1)–(3) is not difficult, and the calculated results are shown inside Figure 6b. The activation energy of No. 4 glass is approximately 189.76 kJ/mol, which is lower than those in other studies used in LTCC [19], so it is easier to crystallize. Variable *n* is related to the mechanism of glass crystallization. When n is close to 1, it is surface crystallization. When n is close to 3, it is volumetric crystallization. The calculated value of n of No.4 glass is 2.325, which is in the range of 1–3. Therefore, the crystallization mode of No.4 glass may be a combination of two mechanisms, and it is more inclined to volumetric crystallization.

**Figure 7:**
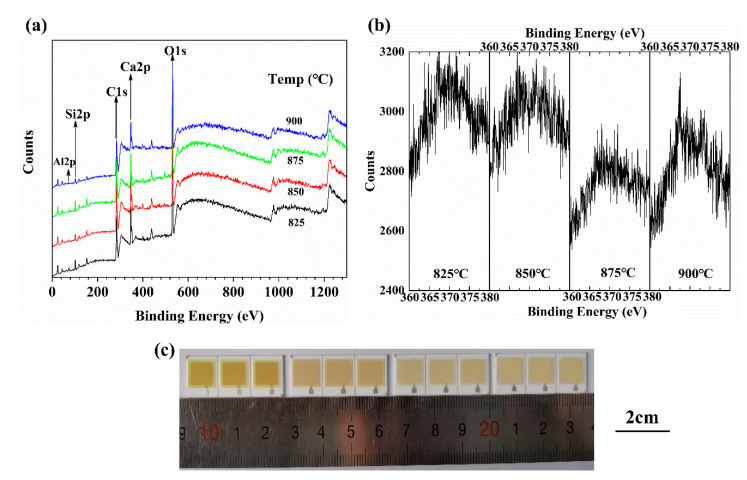
XPS spectra of No. 4 insulating film sintered at 825–900 °C were tested on the surface. (**b**) The XPS magnification near 370 eV of the red dashed circle in (**a**). (**c**) Surface morphology of insulating film sintered at 825–900 °C on Ag film.

In addition, we also found a very interesting phenomenon. With the increase in sintering temperature, the color of the insulation layer changed from dark yellow to light brown (Figure 4c). Many studies have shown that the dielectric layer co-fired with Ag has a similar phenomenon due to the diffusion of Ag into the glass to form a mixture [20,21,22]. Hidekazu found that the discolored yellow mixtures exhibited a large absorption peak near 410 nm [23]. The diffusion of Ag in the insulating film will also deteriorate the performance of the device, so we need to understand the diffusion of Ag in the insulating film. An insulating film, Ag peak was found near 2θ = 64°, and the peak intensity weakened gradually with the increase in temperature and almost disappeared when the temperature was above 875 °C (Figure 5). Figure 7a also shows the XPS spectra of insulating film sintered at 825–900 °C. As expected, with the increase in temperature, the peaks of Al, Si, Ca, and O appear in corresponding positions, and the spectra did not change greatly. However, a small peak is observed near 370 eV. With the increase in temperature, the shape of the peak changes from sharp to broad. Figure 7b is an enlarged view of the peak near 370 eV, which was marked by a red dotted circle in Figure 7a. The binding-energy was concentrated around 368 eV, which is close to Ag 3d_5/2_ [20], indicating that there was Ag diffusion in the insulating layer. However, the peak type is not sharp, so the content of Ag was small. When the temperature was higher than 850 °C, the peak intensity decreased slightly, which indicates that the decrease of Ag content can promote the lightening of color, as shown in Figure 7c.

**Figure 8:**
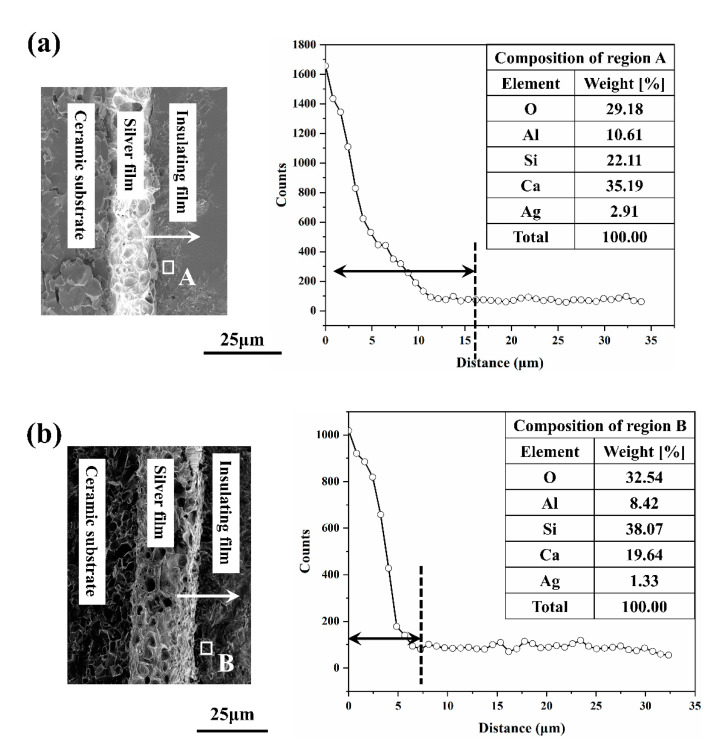
SEM micrograph and EDS curve showing silver diffusion in insulating film sintered at (**a**) 825 °C; (**b**) 875 °C. Table in the figure is EDS data, which showing compositions corresponding to regions A and B.

In order to further observe the diffusion of Ag in the insulating film, the interface was scanned by EDS. Figure 8 shows that with the increase of sintering temperature, the crystallization gradually increases, which reduces the diffusion of Ag in the insulation film. Ag was found on the area of the insulating film near the Ag film, but the content of Ag was lower at high-temperature (region B). With the increase in temperature, the number of crystalline phase increase [21], which has an inhibitory effect on the diffusion of Ag, so the Ag content in the insulating film decreases. Therefore, the reduction of Ag content in the insulation film has a positive effect on reducing the surface yellow color. It is believed that reducing the Ag content in the insulating film will contribute to the electrical properties of the multilayer structure.

### 3.3. Property Variations of Insulating Thick Films

**Figure 9:**
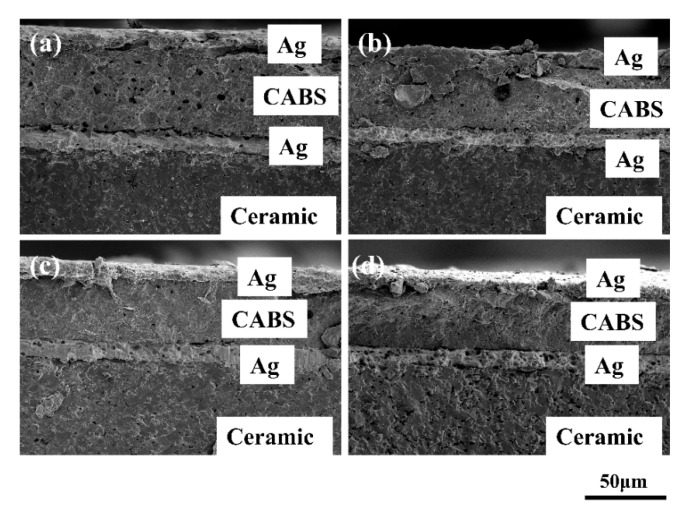
Cross-section of the multilayer structure containing insulating film made of No. 4 glass sintered at: (**a**) 825 °C; (**b**) 850 °C; (**c**) 875 °C; (**d**) 900 °C.

The cross-section of the thick insulating film made of No. 4 glass in the multilayer structure sintered at 825–900 °C for 30 min is shown in Figure 9. The insulation film has been formed, and it is located between the Ag films. At low-temperature, the sintering of the upper Ag layer was not enough, so there were some pores in the insulating film. With the increase of temperature, the sintering of the Ag layer was more sufficient, the pores in the insulation layer became less, and the whole insulating film became more compact, which should have a positive effect on the performance.

**Figure 10:**
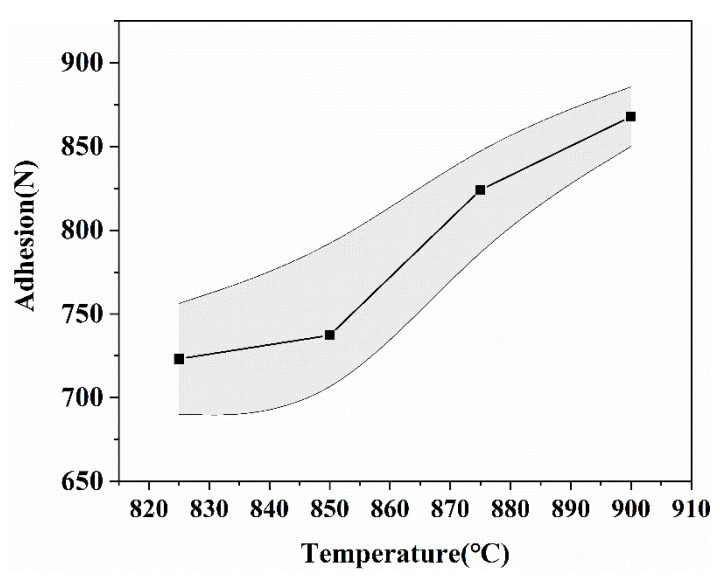
Statistic results of adhesion measurement.

The statistic results of adhesion measurement and electrical parameters of the insulating film in the multilayer structure are shown in Figure 10 and Table 3. Adhesion and electrical parameters were tested at six specimens. In Figure 10, with the increase in temperature from 825 °C to 900 °C, the adhesion strength gradually increased from 750 N to 875 N, and the standard deviation of the test gradually decreased and tended to be stable. With the increase in temperature, the sintering was more sufficient, and the porosity inside the film was reduced, as shown in Figure 9, resulting in a closer bond between the films, thus improving the adhesion. In addition, the pores on the surface of the insulating layer promote the physical occlusion of the upper Ag, so the adhesion had a further improvement.

The electrical parameters of the insulation film were tested by using the test pattern in Figure 1b. The main concern includes the breakdown voltage and dielectric strength of the insulating film, which are key parameters in the evaluation of insulation performance. The electrical parameters of the multilayer structure are described in Table 3. The dielectric constant and dielectric strength were obtained by calculation, and the formula is expressed as follows [6]:

(4) $$\varepsilon_{r}=(d\times C)/(\varepsilon_{0}\times S)$$

(5) $$E_{b}=U_{b}/d$$

where *ε_r_* is the dielectric constant, *d* is the thickness of the insulating film, *C* is the capacity, *ε*_0_ is the dielectric constant of the vacuum, *S* is the area of Ag electrode (top Ag film), *E_b_* is the dielectric strength of the insulating film, and *U_b_* is the breakdown voltage.

Table 3 shows that with the increase in temperature, the dielectric constant presented a slight upward trend, whereas the dielectric loss presented the opposite situation. The dielectric constant and dielectric loss of the insulating film were mainly related to two factors: Ag content in the insulating film and the pores. First, with the increase of temperature, the porosity of insulating film decreased (Figure 9), which makes the dielectric constant gradually increase, and the dielectric loss gradually decreased. In addition, at low-temperature, the Ag content in the insulation layer was greater (Figure 8), which worsened its electrical properties [10]. When the temperature increased, there were more crystalline phases in the insulation layer, and the Ag content decreased, which increased the dielectric constant and reduces the dielectric loss. The two factors were consistent with the change in temperature. With the increase of temperature, the insulating layer was denser, and the Ag content was less, so its electrical performance was better (The dielectric constant increased and the dielectric loss decreased). The increase of dielectric strength was also attributed to the less Ag content and a more compact structure in the insulating film. Therefore, when the temperature was above 875 °C, the dielectric strength was greatly improved. The highest dielectric strength could reach 13.11 kV/mm, which was slightly higher than Heraeus IP9319D (9.90 kV/mm), meeting the packaging needs of power electronics and other devices [6].

## 4. Conclusions

This work demonstrated the characterization of glass insulating thick films with Ag conductors for multilayer packages. The insulating film is made of CaO-Al_2_O_3_-B_2_O_3_-SiO_2_ glass, and the surface is uniform and crack-free when the mass ratio is 35:15:10:40. The glass was screen-printed on the surface of Ag film and sintered above 850 °C to form glass-ceramics. The main crystal phase is CaAl_2_Si_2_O_8_, and the crystallization activation energy was 189.76 kJ/mol. Ag diffused to the inside of the insulating film to make it yellow, and the color change could be lowered by increasing the temperature. The multilayer structure made of glass insulating paste and Ag paste was dense without obvious pores, and the adhesion could reach 875 N. The dielectric constant of the insulating film in the multilayer structure was approximately 5, and the dielectric loss was 0.0011. After sintering at 875 °C, the dielectric strength of the insulating film could reach 13.11 kV/mm, which fully meets the requirements of the complex packaging structure.

The insulating film is very suitable to be used between Ag films to construct multilayer circuits. The main practical application of multilayer structure was to realize the crossing of conductive metal lines on the same ceramic substrate. In some applications, such as power module or light-emitting diode (LED) module, many electronic components are integrated on the smaller size substrate, so they cannot eliminate these crossings just by proper design. Therefore, the multilayer circuit based on reported insulating film and Ag film is very suitable for the power module and LED module, which needs a complex connection between electronic components.

## Figures and Tables

**Table 1:** Raw material ratio of insulating paste.

	Raw Materials	CaCO_3_ (wt %)	Al_2_O_3_ (wt %)	SiO_2_ (wt %)	H_3_BO_3_ (wt %)
Sample					
1		52	18	20	10
2		45	20	25	10
3		38	22	30	10
4		35	15	40	10
5		30	15	45	10

**Table 2:** Description of test specimens.

Film	Paste	Thickness (μm)	Dimensions	
			Electrical Measurement	Adhesion Measurement
Below Ag film	3Focus F14015	41	Square, 8 × 8 mm^2^	Square, 7 × 7 mm^2^
Insulating film	Our own insulating paste	measurement	Square, 10 × 10 mm^2^	Square, 6 × 6 mm^2^
Top Ag film	3Focus F14015	29	Square, 9 × 9 mm^2^	Circle, Φ 3 mm

**Table 3:** Electrical parameters of an insulating film in a multilayer structure.

Electrical Parameter	Sintering Temperature (°C)			
	825	850	875	900
Capacity at 100 kHz (pF)	62.95	87.48	94.68	96.5
Dielectric constant at 100 kHz	3.25	4.52	4.87	4.98
Dielectric loss at 100 kHz	0.0018	0.0012	0.0011	0.0013
Breakdown voltage (V)	361	388	485	472
Dielectric strength (kV/mm)	9.76	10.49	13.11	12.76

## Data Availability

Data available in a publicly accessible repository.
